# The Pro-Apoptotic and Pro-Inflammatory Effects of Calprotectin on Human Periodontal Ligament Cells

**DOI:** 10.1371/journal.pone.0110421

**Published:** 2014-10-22

**Authors:** Yunfei Zheng, Jianxia Hou, Lei Peng, Xin Zhang, Lingfei Jia, Xian'e Wang, Shicheng Wei, Huanxin Meng

**Affiliations:** 1 Department of Oral and Maxillofacial Surgery, Peking University School and Hospital of Stomatology, Beijing, P.R. China; 2 Department of Periodontology, Peking University School and Hospital of Stomatology, Beijing, P.R. China; 3 Department of Stomatology, Beijing Friendship Hospital, Capital Medical University, Beijing, P.R. China; 4 Department of Stomatology, Peking Union Medical College Hospital, Chinese Academy of Medical Science, Beijing, P.R. China; 5 Laboratory of Molecular Biology, Peking University School and Hospital of Stomatology, Beijing, China; University of Toronto, Canada

## Abstract

Calprotectin, a heterodimer of S100A8 and S100A9 subunits, is associated with inflammatory disorders such as rheumatoid arthritis and cystic fibrosis. Although calprotectin levels are increased significantly in the gingival crevicular fluid (GCF) of periodontitis patients, its effects on periodontal ligament cells (PDLCs) remain largely unknown. The aim of this study was to evaluate calprotectin levels in the GCF of generalized aggressive periodontitis (AgP) patients and to investigate the effects of recombinant human calprotectin (rhS100A8/A9) and its subunits (rhS100A8 and rhS100A9) in PDLCs. Both the concentration and amount of crevicular calprotectin were significantly higher in the AgP group compared with healthy controls. In addition, the GCF calprotectin levels were correlated positively with clinical periodontal parameters including bleeding index, probing depth, and clinical attachment loss. rhS100A8/A9 promoted cell apoptosis, whereas rhS100A8 and rhS100A9 individually exerted little effect on apoptosis in PDLCs. rhS100A9 and rhS100A8/A9 increased the activation of nuclear factor-κB (NF-κB) by promoting the nuclear translocation of p65 in PDLCs, subsequently inducing expression of the pro-inflammatory cytokines IL-6, IL-8, TNFα, and COX2. Treatment with an NF-κB inhibitor partially reversed the rhS100A9- and rhS100A8/A9-induced upregulation of the pro-inflammatory cytokines. rhS100A9, and not rhS100A8, was mainly responsible for the pro-inflammatory role of calprotectin. Collectively, our results suggest that calprotectin promotes apoptosis and the inflammatory response in PDLCs via rhS100A9. These findings might help identify novel treatments for periodontitis.

## Introduction

Periodontitis is an infectious disease that affects the tissues supporting the teeth and leads to eventual tooth loss [1]. Apart from the type associated with systemic conditions, periodontitis can be divided into two broad categories; chronic periodontitis, which occurs primarily in adults and progresses relatively slowly, and aggressive periodontitis, a more aggressive form that may occur in adolescents. Current knowledge regarding the pathogenesis of periodontal disease suggests that its central cause is an imbalance in the host-parasite relationship. The host inflammatory response also plays a role. A large number of cytokines and other effector molecules released by resident and migrating cells contribute to the destruction of soft and hard tissues seen in periodontitis [2].

Calprotectin is a heterodimeric calcium-binding protein consisting of S100A8 and S100A9 subunits [3]. It is expressed constitutively in neutrophils [4], monocytes [4], keratinocytes [5]. It also plays role in epithelial cells [6] and several different cancer cells [3], [7], [8]. Calprotectin is estimated to constitute ∼40% of the total cytosolic proteins in polymorphonuclear neutrophil [4], [7]. It is released during inflammatory events, either after cell death or via an active secretary mechanism [9]. Calprotectin exerts antiproliferative and antitumoral effects [10], [11]. In addition, it acts not only as a chemoattractant [12], but also as a pro-inflammatory factor that exerts cytokine-like activities. For example, it can bind to Toll-like receptor 4 (TLR-4) or the advanced glycation end product receptor to activate the intracellular signaling pathways including the mitogen-activated protein kinase and nuclear factor-κB (NF-κB) pathways [13], [14]. Increased levels of calprotectin in extracellular fluid were reported in numerous inflammation-associated pathological conditions, such as rheumatoid arthritis, Sjögren's syndrome, Crohn's Diseas, colorectal cancer and periodontitis [7]. The levels of calprotectin in the gingival crevicular fluid (GCF) of patients with gingivitis and periodontitis were reported, and its concentration was correlated with clinical factors such as probing depth (PD), bleeding on probing (BOP), and gingival index [15], [16]. Conversely, non-surgical therapy using antibiotics decreased local calprotectin levels [17].

Interestingly, human S100A8 is a potent and specific autocrine chemotactic factor in periodontal ligament cells (PDLCs); therefore, it might be an attractive therapeutic candidate for the treatment of periodontal disease [18], [19]. However, the concentration of S100A8 that stimulates the chemotactic activity of PDLCs is far lower than the concentration *in vivo*. PDLCs reside in the periodontal ligament space and play a key role in forming the periodontal ligament and its fibers by secreting extracellular matrix components such as collagen and mineralized tissue [20]. PDLCs also play a role in the production of various pro-inflammatory cytokines, thereby participating in the development of periodontal inflammation.

In recent years the regenerative potential of PDLCs in tissue repair has received increasing attention [21]. Given the importance of PDLCs in maintaining of the integrity of periodontal tissue, the effects of calprotectin and its subunits in PDLCs, other than their chemotactic activity, is worthy of investigation. To determine a potential correlation between calprotectin levels and periodontitis and to examine the mechanism of calprotectin's influence on the behavior of PDLCs, calprotectin levels in the GCF of patients with generalized aggressive periodontitis (AgP) were evaluated and the pro-apoptotic and pro-inflammatory effects of recombinant S100A8, S100A9, and S100A8/A9 in PDLCs were investigated.

## Materials and Methods

### Ethics statement

The Ethics Committee of Peking University School of Stomatology approved the study protocol, and all clinical specimens were obtained from patients who provided written informed consent to use their tissues for research purposes (approval number PKUSSIRB-2011007).

### Study population

A total of 40 participants were included in the study. Of these, 20 patients <36 years old with generalized aggressive periodontitis were recruited from the Department of Periodontology, Peking University Hospital of Stomatology, China. The diagnostic criteria for generalized aggressive periodontitis were defined according to the classification proposed at the International Workshop for the Classification of Periodontal Diseases and Conditions in 1999 [22]. The details of inclusion criteria were as follows: (1) patients are affected at <35 years of age; (2) at least eight teeth had a probing depth (PD) >6 mm, three or more of which were not first molars or incisors; and (3) radiographs showed alveolar bone loss. In addition, 20 periodontally healthy controls were selected from the staff and students at the School of Stomatology; they were included if they meet the following criteria as previously described [22]: (1) PD ≤3 mm; (2) bleeding on probing (BOP) was <10%; (3) no attachment loss (AL); and (4) no bone loss visible on radiographs. All participants were non-smokers and no participants had a concomitant systemic disease or received antibiotics within the 4 months prior to the study. Patients who had received periodontal therapy within the previous year and females who were post-menopausal, pregnant, in lactation or taking oral contraceptive drugs were excluded from the study. The purpose of the study and the procedures involved were explained to all participants in advance and written informed consent was obtained.

### Clinical examination

All subjects underwent a clinical periodontal examination including PD, BI, and CAL using a Williams periodontal probe at six sites of each tooth to determine their clinical periodontal status [22]. A set of full-mouth periapical radiographs was also taken from each subject.

### Collection of GCF samples

Two sites of each subject were selected for GCF sampling, one from the mesial buccal site of the right maxillary central incisor and the other from the mesial buccal site of the left mandibular first molar. GCF samples were collected using a paper strip (Whatman, Maidstone, U.K.) from healthy gingival crevices (PD ≤2 mm, BI  = 0, CAL  = 0 mm) for periodontally healthy controls and from periodontal pockets (PD ≥4 mm, BI ≥1, CAL ≥1 mm) in patients with generalized aggressive periodontitis. Supragingival plaques were removed from the interproximal surfaces using a sterile curette prior to the sampling. Paper strips were then inserted carefully into the crevice until mild resistance was felt and left there for 30 seconds.

### Measuring calprotectin levels

GCF samples were eluted from the strips by placing them in 300 µl of phosphate buffered saline (PBS). The calprotectin levels were then assayed using commercially available ELISA kits according to the manufacturer's instructions (Phicaltest; Calpro AS, Oslo, Norway). The amount of calprotectin in each sample was calculated based on the dilutions as described previously [23].

### Production of recombinant human S100A8 (rhS100A8) and S100A9 (rhS100A9)

The methods used to produce and purify the recombinant proteins were described previously [24]. Briefly, the hS100A8 and hS100A9 plasmids (kind gifts from Dr. Tessier, Infectious Diseases Research Center, Laval University Hospital Center, Québec, Canada) [24] were transformed into *Escherichia coli* BAL21 (DE3) and their expression was induced using isopropyl β-D-thiogalactosidein (Sigma-Aldrich, St. Louis, MO, USA). Next, a nickel column and a polymyxin B-agarose column (Pierce, Rockford, IL, USA) were used to purify the recombinant His-tagged proteins. The endotoxins contamination was <1 pg/µg protein, as measured using a Limulus amebocyte assay (Sigma-Aldrich). Recombinant proteins were analyzed on SDS-PAGE gels and stained using Coomassie brilliant blue. As shown in Figure S1, rhS100A8 and rhS100A9 migrated to their expected molecular masses and were free of contaminating bacteria-derived proteins after purification. Equimolar amounts of rhS100A8 and rhS100A9 were mixed to generate rhS100A8/A9 in the presence of 1.3 mM Ca^2+^ according to Vandal et al [25].

### Cell culture

Primary normal human periodontal ligament cells were obtained from explant cultures using methods described previously [26]. Briefly, periodontal ligament explants were obtained from the middle third of premolar roots extracted for orthodontic treatment and were minced into smaller portions. The cells were maintained in Dulbecco's modified Eagle medium (DMEM; Gibco, Gaithersburg, MD, USA) supplemented with 10% heat-inactivated fetal bovine serum (Hyclone, Logan, UT, USA) in tissue culture flasks (Corning, NY, USA). Cells from passages four to eight were used all experiments.

### Apoptosis analysis

To detect cell apoptosis, an annexin V-fluorescein isothiocyanate (FITC) kit (Roche, Mannheim, Germany) was used according to the manufacturer's protocol. Briefly, PDLCs were treated with rhS100A8, rhS100A9 or rhS100A8/A9, incubated for 48 h, trypsinized, and then re-suspended in the binding buffer containing 5 µL of annexin V-FITC and 4 µg/mL of propidium iodide after incubation in the dark at room temperature for 10 min. Apoptosis were then analyzed using fluorescence-activated cell sorting (Beckman Coulter, Fullerton, CA, USA). At least 10,000 cells per sample were analyzed and annexin-V-FITC-positive cells were regarded to be apoptotic.

The morphology of PDLCs treated with calprotectin were evaluated using fluorescence microscopy after staining with 4′,6-diamidino-2-phenylindole (DAPI). Approximately 5×10^4^ PDLCs per well of a 24-well plate were incubated with rhS100A8, rhS100A9 or rhS100A8/A9 at a final concentration of 10^−6^ M for 48 h. They were then fixed with 4% paraformaldehyde (Sigma-Aldrich), stained with DAPI and visualized using a fluorescence microscope as described previously [27].

### RNA extraction and real-time PCR analysis

Subconfluent cell monolayers were serum-depleted overnight in DMEM and were then treated with rhS100A8, rhS100A9 or rhS100A8/A9 alone or in combination with the NF-κB specific inhibitor pyrrolidine dithiocarbamate (PDTC; Sigma-Aldrich) for 12 h. Total RNA was isolated using RNA isolation reagent TRIzol (Invitrogen, Carlsbad, CA, USA) according to the manufacturer's instructions, and the concentration was determined spectrophotometrically. First-strand cDNA was synthesized using a first-strand cDNA synthesis kit (MBI Fermentas, Inc., Burlington, ON, Canada) using oligo dT primers. The primers used were as follows: human IL-1β forward 5′-ATG GCT TAT TAC AGT GGC A-3′ and reverse 5′-GTA GTG GTG GTC GGA GAT T-3′, human IL-6 forward 5′-GTG AGG AAC AAG CCA GAG C-3′ and reverse 5′-TAC ATT TGC CGA AGA GCC-3′, human IL-8 forward 5′-TTT TGC CAA GGA GTG CTA AAG A-3′ and reverse 5′-AAC CCT CTG CAC CCA GTT TTC-3′, human TNFα forward 5′-CGA GTG ACA AGC CTG TAG CC-3′ and reverse 5′-TGA AGA GGA CCT GGG AGT AGA T-3′, human COX2 forward 5′-CTG GCG CTC AGC CAT ACA G-3′ and reverse 5′-ACA CTC ATA CAT ACA CCT CGG T-3′, human GAPDH forward 5′-ATG GGG AAG GTG AAG GTC G-3′ and reverse 5′-GGG GTC ATT GAT GGC AAC AAT A-3′. Real-time PCR was performed using a 7500 Fast Real-time RT-PCR System (Applied Biosystems, Foster City, CA) according to the manufacturer's instructions. The relative expression of the targed gene was then normalized to GAPDH expression using 2^−ΔΔCt^ method.

### ELISA

The concentrations of IL-6 and IL-8 in cell culture supernatants were determined using ELISA (Gersion, Beijing, China) according to the manufacturer's instructions. Briefly, the supernatants of PDLCs treated with 10^−6^ M rhS100A8, rhS100A9 and rhS100A8/A9 were added to the ELISA plates incubated with antibodies, aspirated and then washed with washing buffer. The optical density at 450 nm was then measured.

### Immunofluorescent analysis of p65

PDLCs were seeded on glass slides at a density of 5×10^4^ cells/mL, and treated with rhS100A8, rhS100A9 or rhS100A8/A9 for 6 h. Immunofluorescent analysis of the p65 was then performed as described previously [28]. Briefly, cultures were fixed in 4% paraformaldehyde, permeabilized with 0.1% Triton X-100 and blocked with 10% goat serum. After incubation overnight at 4°C in a humid chamber with antibodies against the 65 kDa subunit of NF-κB (1∶100) (Beyotime, Jiangsu, China), slides were incubated with tetramethyl rhodamine isothiocyanate-conjugated goat anti-rabbit secondary antibodies (1∶200) (Chemicon, Hofheim, Germany) for 30 min at room temperature; 5 µg/mL DAPI was used for conterstaining. Finally, the slides were embedded in mounting medium and the fluorescence signals were measured using a Zeiss laser scanning microscope (LSM 510; Carl Zeiss, Jena, Germany).

### EMSA

Nuclear PDLC extracts were prepared and EMSAs were performed as described previously [28]. 3′-Biotin-labeled and unlabeled probes containing the NF-κB consensus binding site (5′-AGT TGA GGG GAC TTT CCC AGG C-3′) were synthesized, and the binding reaction was performed using a Light Shift Chemiluminescent EMSA Kit (Pierce, Rockford, IL, USA).

### Statistical analysis

Statistical analyses were performed using SPSS PASW version 18. Distribution of the variables was tested using the Shapiro-Wilk test. Continuous normally distributed variables were presented as means ± standard deviations (SD). The median (lower to upper quartile) was used to describe non-normally distributed data. Differences for age and calprotectin levels in the GCF between groups were analyzed using Student's t-test, whereas differences for clinical parameters between groups were analyzed using Mann-Whitney U-test. Gender was analyzed using chi-square test. Spearman's correlation tests were used to identify the associations between local clinical variables and the calprotectin levels. Pearson's correlation coefficient is used to identify the association between the age and GCF calprotectin level. Cell culture experiments were performed at least in triplicate, and the differences among groups were analyzed using one-way ANOVA. A two-tailed *p* value <0.05 was considered to indicate statistical significance.

## Results

### Increased GCF calprotecin levels in the AgP group

The demographic characteristics of the 40 study participants are summarized in Table 1. There were no significant differences in age or gender between the two groups. Generally, patients in the AgP group had more severe clinical indices with respect to BI, PD and CAL. Both the concentration and amount of GCF calprotectin were significantly higher in the AgP group compared with the control group (2,332±383.200 µg/mL *vs*. 471±51.120 µg/mL, and 4,378±741.4 ng *vs*. 87.82±12.38 ng, respectively; *p*<0.05). The calprotectin levels in the GCF were moderately positively correlated with clinical periodontal parameters including BI, PD and CAL (*p*<0.05, Table 2). There was no association with age or gender.

**Table 1:** Demographic and clinical variables.

variables	Healthy group (n = 20)	AgP group (n = 20)	*p* value
Age(years)^a^	24.40±0.3434	26.80±1.615	0.1543
Gender(n; male/female)^b^	10/10	6/14	0.197
BI^c^	2 (1–3)	4 (3–4)	**<0.01**
PD(mm)^c^	2.5 (2.0–3.0)	7.0 (5.3–7.0)	**<0.01**
CAL(mm)^c^	0	6.0 (5.0–7.75)	**<0.01**
CPT Conc. in GCF(µg/mL) ^a^	471±51.120	2332±383.200	**<0.01**
CPT amount in GCF(ng)^a^	87.82±12.38	4378±741.4	**<0.01**

Data are given as Mean ± SD or number of subjects, or median (lower-upper quartile).

Between-group comparisons were performed using the ^a^t-test, ^b^chi-square test, or ^c^Mann-Whitney U-test.

Values in bold indicate a statistically significant difference (p<0.01).

AgP, generalized aggressive periodontitis; BI, bleeding index; PD, probing depth; CAL, clinical attachment loss; CPT Conc., calprotectin concentration; CPT amount, the amount of calprotectin; GCF, gingival crevicular fluid.

**Table 2:** Correlations between crevicular calprotecin levels and the variables evaluated in the study cohort (n = 40).

Variables	crevicular calprotectin conc.		crevicular calprotectin amount	
	r	*p*-value	r	*p*-value
Demographic parameters				
age	0.03373	0.8363^a^	0.2539	0.1138^a^
gender	−0.07515	0.6449^b^	0.2520	0.1167^b^
Clinical parameters				
Mean BI	0.5503	**<0.01^b^**	0.6271	**<0.01^b^**
Mean PD	0.5010	**<0.01^b^**	0.7246	**<0.01^b^**
Mean CAL	0.5350	**<0.01^b^**	0.7659	**<0.01^b^**
CPT conc. in GCF	—	—	0.7626	**<0.01^a^**
CPT amount in GCF	0.7626	**<0.01^a^**	—	—

Correlation analysis was performed using ^a^Pearson's rank correlation analyses or ^b^Spearman's rank correlation analyses.

Bold numbers indicate a statistically significant difference (p<0.01).

BI, bleeding index; PD, probing depth; CAL, clinical attachment loss; CPT Conc., calprotectin concentration; CPT amount, the amount of calprotectin; GCF, gingival crevicular fluid.

### Pro-apoptotic activity of rhS100A8, rhS100A9 and rhS100A8/A9

**Figure 1:**
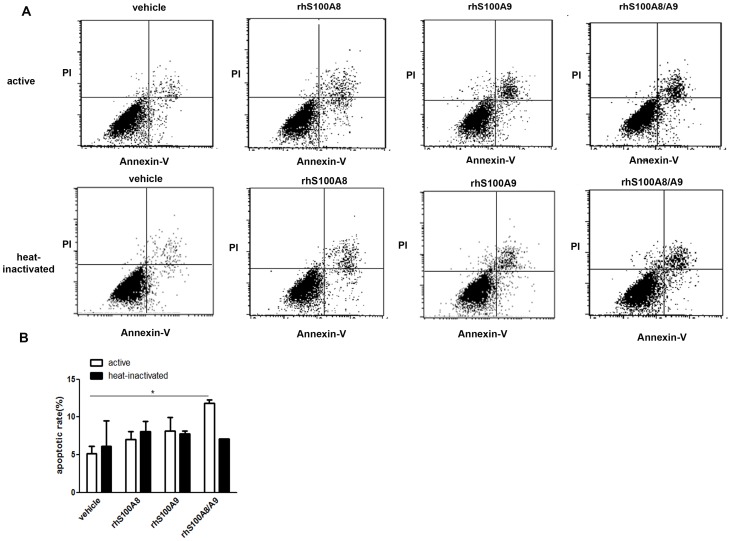
Analysis of PDLC apoptosis using annexin V/PI staining. PDLCs were treated with 10^−6^ M rhS100A8, rhS100A9, rhS100A8/A9 or heat-inactivated rhS100A8, rhS100A9, rhS100A8/A9 for 48 h. Apoptosis was then assessed using flow cytometry after annexin V/PI staining. The fluorescence intensity of 10,000 cells was measured and the number of annexin-V-FITC-positive cells was calculated. Statistical analysis revealed that there were significantly more apoptotic cells in the rhS100A8/A9-treated group (10.2475%±2.019%, *p*<0.05) compared with the control group (5.653%±2.914%). There were no differences between the rhS100A8- and rhS100A9-treated groups. * *p*<0.05 *vs*. the control group.

Flow cytometry revealed that the number of annexin V-positive PDLCs increased significantly upon exposure to rhS100A8/A9 for 48 h, whereas only ∼5% of cells were annexin V-positive in the vehicle group. Fewer apoptotic cells were detected in the presence of rhS100A8 or rhS100A9 compared with rhS100A8/A9 (Figure 1).

**Figure 2:**
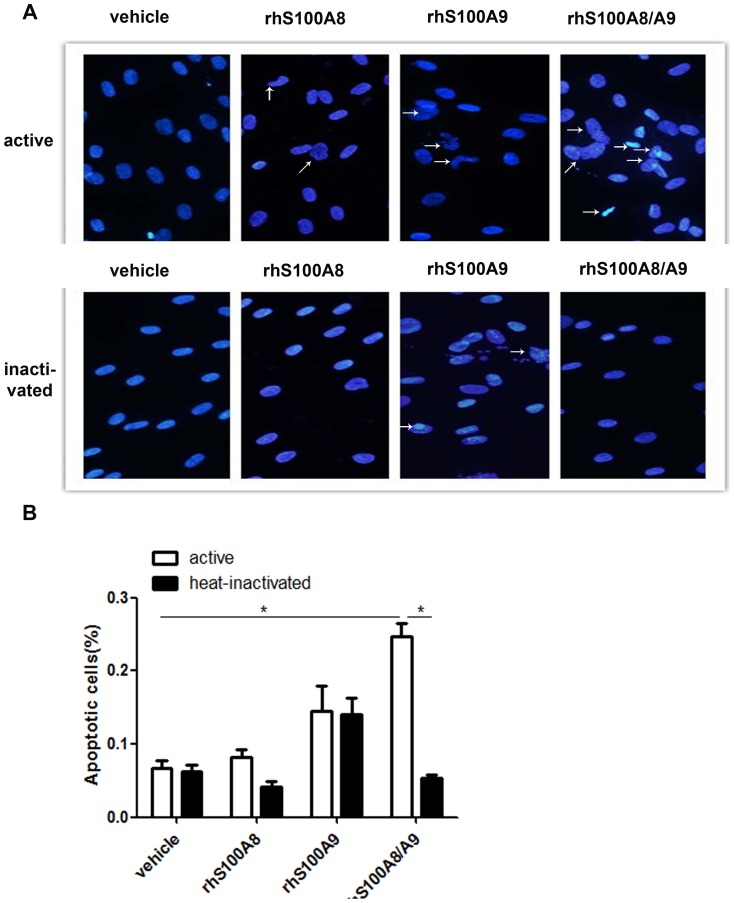
Morphology of PDLCs after DAPI staining. The morphological changes of PDLCs treated with rhS100A8, rhS100A9, rhS100A8/A9, or heat-inactivated rhS100A8, rhS100A9, or rhS100A8/A9 were evaluated by fluorescence microscopy after DAPI staining. Viable cells displayed a normal-sized nucleus and blue fluorescence, whereas apoptotic cells exhibited condensed chromatin and fragmented nuclei. Treatment with rhS100A8/A9 increased the percentage of apoptotic cells significantly compared with the control (24.5%±5.1% *vs*. 6.7%±3.1%, *p*<0.05) and heat-inactivated rhS100A8/A9 (24.5%±5.1% *vs*. 5.273%±1.14%, *p*<0.05). Fewer apoptotic cells were observed in rhS100A8-treated cultures whereas more apoptotic cells were present in rhS100A9-treated cells. * *p*<0.05 *vs*. the control or heat-inactivated groups.

Morphological changes in PDLCs treated with rhS100A8, rhS100A9 and rhS100A8/A9 were analyzed using fluorescence microscopy following DAPI staining (Figure 2). Viable cells exhibit a normal-sized nucleus and blue fluorescence, whereas apoptotic cells contain condensed chromatin and fragmented nuclei. After treatment with rhS100A8/A9 for 48 h, the number of apoptotic cells increased significantly (24.5%±5.1% *vs.* 6.7%±3.1%, *p*<0.05). Fewer apoptotic cells were observed in rhS100A8-treated cultures compared with rhS100A9-treated cultures. Because the recombinant S100A proteins used in these studies were derived from *E. coli* extracts, it is possible that the response was due to trace contamination with bacterial LPS. To exclude this possibility, an aliquot of the recombinant protein was heated at 100°C for 10 min to inactivate the protein; this abrogated its pro-apoptotic activity.

### rhS100A8, rhS100A9 and rhS100A8/A9 induce pro-inflammatory cytokines

**Figure 3:**
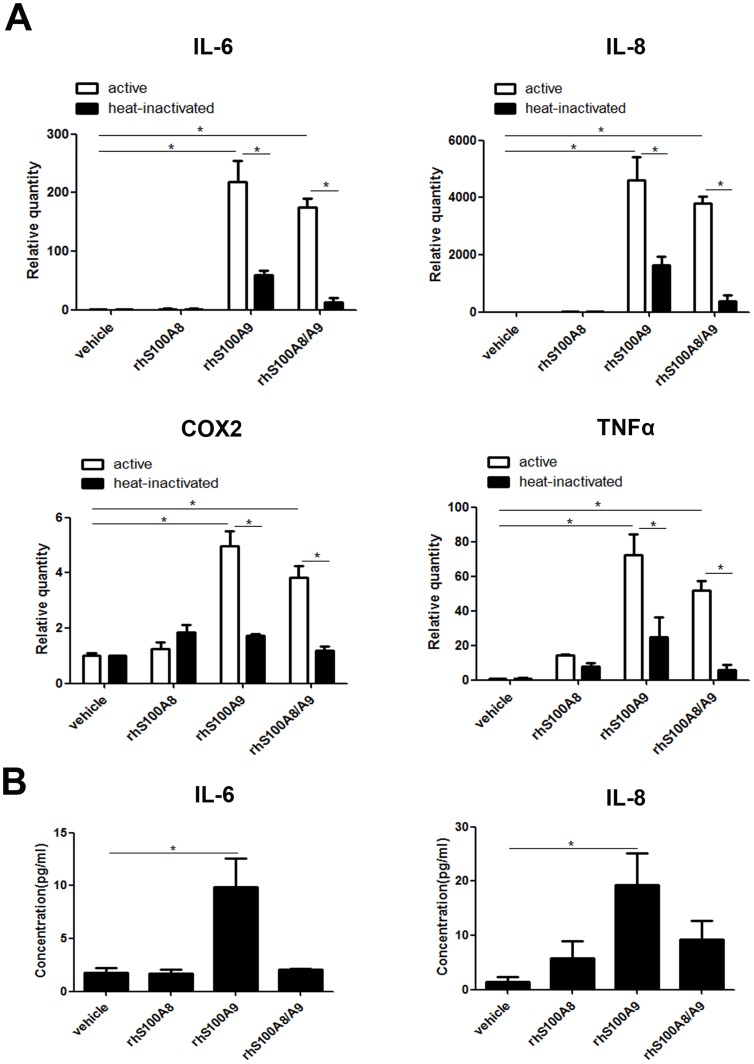
rhS100A8, rhS100A9 and rhS100A8/9 upregulate pro-inflammatory cytokines in PDLCs. (A). PDLCs were treated with rhS100A8, rhS100A9 or rhS100A8/A9 for 12 h, and the mRNA levels of cytokines were analyzed. rhS100A9 and rhS100A8/A9 upregulated the expression of IL-6, IL-8, TNFα, and COX2 significantly compared with rhS100A8 and the control group (*p*<0.05). (B). PDLCs were treated with rhS100A8, rhS100A9 or rhS100A8/A9 for 12 h, and the protein levels of IL-6 and IL-8 in PDLCs culture supernatants were detected using ELISA. rhS100A9 promoted the protein expression of IL-6 and IL-8 significantly (*p*<0.05). * *p*<0.05 *vs*. the control or heat-inactivated groups.

The mRNA levels of IL-6, IL-8, TNFα and COX2 were increased significantly in cells treated with rhS100A9 and rhS100A8/A9 compared with the vehicle and heat-inactivated groups (Figure 3A). IL-6 and IL-8 were the most responsive genes, both upregulated several hundred-fold. Although these genes were also upregulated by rhS100A8, the levels were lower than those induced by rhS100A9 or rhS100A8/A9. Heat-inactivated rhS100A9 and rhS100A8/A9 failed to upregulation IL-6, IL-8, TNFα or COX2, which excluded the possibility of trace LPS contamination. rhS100A9 also increased the protein expression of IL-6 and IL-8 significantly (*p*<0.05; Figure 3B).

### rhS100A9 and rhS100A8/A9 activate NF-κB

**Figure 4:**
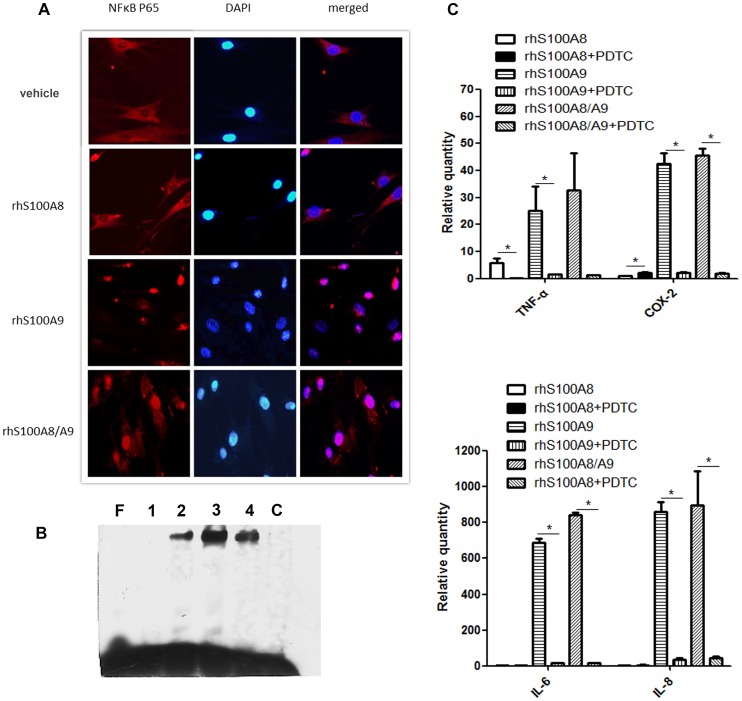
rhS100A8, rhS100A9 and rhS100A8/9 increase NF-κB signaling in PDLCs. (A). PDLCs were treated with rhS100A8, rhS100A9 or rhS100A8/A9 for 6 h, fixed, and then incubated with NF-κB p65 antibodies. The translocation of p65 was observed in cells treated with rhS100A9 and rhS100A8/A9, whereas p65 remained mainly in the cytoplasm of cells treated with rhS100A8. (B). NF-κB activation was assessed using EMSA 12 h after treatment with rhS100A8, rhS100A9, or rhS100A8/A9. Compared with lane F, which contains oligonucleotides without nuclear extracts, a band representing binding was visible after the addition of PDLCs nuclear extracts from cells treated with rhS100A8 (lanes 2), rhS100A9 (lane 3), or rhS100A8/A9 (lane 4). A competition assay was then used to confirm the binding specificity (lane C). The addition of rhS100A8 attenuated the rhS100A9-induced DNA-binding activity of NF-κB. (C). PDTC reversed rhS100A9- and rhS100A8/A9-induced cytokines upregulation. * *p*<0.05 *vs*. the control or heat-inactivated groups.

To determine the mechanism for the pro-inflammatory effects of rhS100A9 and rhS100A8/A9, indirect immunohistofluorescence was performed to detect the nuclear translocation of NF-κB-p65. The presence of red fluorescence in the nucleus, which is suggestive of NF-κB translocation, typically indicates its functional activation because it is otherwise retained in the cytoplasm as inactive complexes with IκB proteins. In untreated PDLCs, p65 was localized almost exclusively in the cytoplasm. In contrast, rhS100A9 and rhS100A8/A9 induced the nuclear translocation of p65, as indicated by the pink nuclear signal in the merged images caused by the overlapping blue (DAPI staining) and red fluorescence signals (NF-κB-p65 subunit) in the nucleus (Figure 4A). rhS100A8 did not stimulate significant translocation of NF-κB-p65, although some small patches of positive NF-κB staining were present in the nuclei.

To further assess the effects of rhS100A8, rhS100A9 and rhS100A8/A9 on NF-κB signaling, oligonucleotides containing the NF-κB-binding sequences were reacted with nuclear extracts. As shown in Figure 4B, a DNA-protein complex was visible in the samples incubated with the nuclear extracts (lanes 2, 3 and 4) compared with the oligonucleotides without the nuclear extracts (lanes 1). A competition assay was then used to verify the binding specificity. The DNA-binding activity of NF-κB in the rhS100A9-treated group was increased compared with the rhS100A8- and rhS100A8/A9-treated groups. The activation of NF-κB signaling was confirmed by the addition of PDTC, an NF-κB inhibitor, which attenuated the rhS100A9- and rhS100A8/A9-induced cytokines upregulation (Figure 4C).

## Discussion

The present study revealed that calprotectin levels were significantly higher in the GCF of AgP patients compared with the control group, which is consistent with the previous studies [15], [29], [30]. Therefore, local calprotectin levels have the potential to be used as a diagnostic marker of periodontitis. However, the biological function of calprotectin in PDLCs and the mechanism by which calprotectin contributes to the pathogenesis of periodontitis remain unclear. Therefore, the biological effect of rhS100A8, rhS100A9 and rhS100A8/A9 in PDLCs was measured in the current study.

S100A8/A9 was reported to have broad apoptosis-inducing activity in various cells including cancer cells such as AGS [31], HaCaT keratinocytes [32], EL-4 lymphoma cells [33], colon carcinoma cells [34], and normal cells like HMEC-1 cells [10]. The apoptosis-inducing effects of rhS100A8, rhS100A9 and rhS100A8/A9 were examined in PDLCs *in vitro* and data revealed that rhS100A8/A9 had greater apoptosis-inducing effects than did rhS100A8 or rhS100A9 alone. Consistent with this, cytotoxic effects of rhS100A8/A9 in EL-4 cells were detected with relatively low concentrations in a standard medium, whereas rhS100A8 and S100A9 caused only marginal cytotoxicity at even higher concentrations [33]. To verify whether the observed pro-apoptotic effects of calprotectin were specific, the cytotoxic activity of heat-inactivated calprotectin was also examined to exclude the possible effects of trace LPS contamination. Heat-inactivating calprotecin abrogated its pro-apoptotic effects in PDLCs. However, fewer PDLCs underwent apoptosis after treatment with rhS100A8/A9 in the current study compared with the previous study in tumor cells. Because cells exhibit variable sensitivity to S100A8/A9 [34], this discrepancy might be because tumor cells are more sensitive to calprotectin, or because of the eukaryotic origin of the protein used in previous studies. Clinically, the pro-apoptotic activity of S100 proteins toward tumor cells raises the possibility that they could be used to treat tumors. However, the apoptosis-inducing activities of calprotectin in normal PDLCs might contribute to periodontal tissue destruction and delayed tissue repair.

Anatomically, periodontal ligament cells are in close proximity to infiltrating inflammatory cells [35]; therefore they are readily accessible to the promoters of inflammation. To determine whether S100 proteins also exacerbate inflammation, the effects of rhS100A8, rhS100A9 and rhS100A8/9 on the expression of several pro-inflammatory cytokines in PDLCs were investigated. Data revealed that treatment with rhS100A9, and rhS100A8/A9 upregulated the expression of IL-6, IL-8, TNFα and COX2 in PDLCs. Importantly, treatment with rhS100A9 and rhS100A8/A9 resulted in the robust expression of IL-6 and IL-8. IL-6 induces osteoclastogenesis [36] and IL-8 functions as a chemoattractant for neutrophils [37]; therefore, their induction leads to further bone destruction and neutrophils infiltration. Furthermore, rhS100A9 and rhS100A8/A9 promoted the activation of NF-κB signaling significantly in the current study, which might explain the upregulation of pro-inflammatory cytokines. Consistent with this, a previous study revealed that S100A8/A9 enhanced the activation of NF-κB by binding to advanced glycation end product receptors and Toll-like receptors [38]. Although both S100A8 and S100A9 induced the NF-κB-dependent transcriptional activity [39], [40], S100A8 induced only mild activation of NF-κB signaling in the current study.

The pro-inflammatory effects of rhS100A9 and rhS100A8/A9 in the current study were significantly greater than those of rhS100A8. Although there is a general consensus that the function of S100A8 or S100A9 is dependent on heterodimer formation, increasing evidence suggests that each subunit alone might exert specific effects. The present study revealed that treatment with rhS100A9 induced more apoptotic cells and a more significant upregulation of pro-inflammatory cytokines compared with rhS100A8. This suggests that S100A9 plays a more important role in the pro-apoptotic and pro-inflammatory actions of rhS100A8/A9 than does S100A8. Consistent with this, S100A9 exerts pro-inflammatory effects by triggering NF-κB activation in human THP1 cells, which is dependent on TLR-4 [13]. Because the effect of rhS100A9 was reversed partly by rhS100A8 as observed in the rhS100A8/A9 group, it is possible that the effects of S100A9 are neutralized in part by S100A8. Other studies support this hypothesis. For example, the S100A9-mediated enhanced integrin-mediated adhesion was inhibited by S100A8 [41], and the addition of S100A8 abrogated the candidastatic effects of S100A9 [42]. Recently, Tessier et al demonstrated that treatment with anti-S100A9 antibodies inhibited the amplified immune response and helped to preserve tissue integrity in a murine arthritis model [43]. Therefore, the protective role of anti-S100A9 might facilitate the treatment of periodontitis; ongoing *in vivo* trials using anti-S100A9 might support this.

In conclusion, rhS100A8/A9 exerted PDLCs apoptosis, whereas rhS100A8 or rhS100A9 were less effective. In addition, rhS100A9 and rhS100A8/A9 amplified the pro-inflammatory cytokine response by activating NF-κB signaling in PDLCs; rhS100A8 was less potent. These results suggest that reducing local S100A9 or S100A8/A9 levels might have therapeutic potential for periodontitis.

## Supporting Information

Figure S1 **SDS-PAGE analysis of recombinant S100A8 and S100A9 stained with Coomassie brilliant blue.** Recombinant human S100A8 (lane 3) and S100A9 (lane 5) were expressed after induction with β-D-thiogalactoside but were absent in uninduced lysate (lane 2). Purified rhS100A8 and rhS100A9 were free from other contaminants (lanes 4 and 6).
(TIF)
