# Supplementary figures and images for: The Pro-Apoptotic and Pro-Inflammatory Effects of Calprotectin on Human Periodontal Ligament Cells
Source: PLoS One. 2014 Oct 22;9(10):e110421. doi: 10.1371/journal.pone.0110421 (PMC4206420; doi:10.1371/journal.pone.0110421)

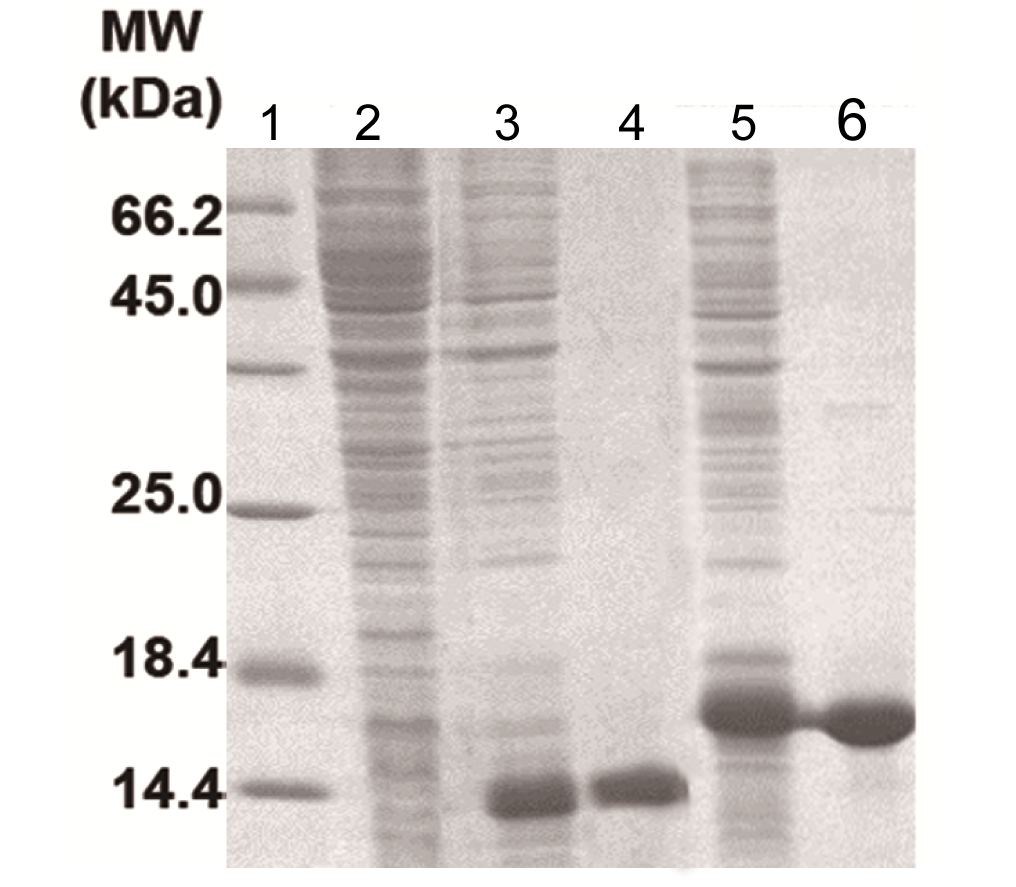

Supplement: Figure S1 — SDS-PAGE analysis of recombinant S100A8 and S100A9 stained with Coomassie brilliant blue. Recombinant human S100A8 (lane 3) and S100A9 (lane 5) were expressed after induction with β-D-thiogalactoside but were absent in uninduced lysate (lane 2). Purified rhS100A8 and rhS100A9 were free from other contaminants (lanes 4 and 6). (TIF) [file pone.0110421.s001.tif]
